# A Sociotechnical Framework for Governing Climate Engineering

**DOI:** 10.1177/0162243915591855

**Published:** 2015-06-24

**Authors:** Rob Bellamy

**Affiliations:** 1 Institute for Science, Innovation and Society, University of Oxford, Oxford, UK

**Keywords:** engagement, intervention, politics, power, governance, appraisal

## Abstract

Proposed ways of governing climate engineering have most often been supported by narrowly framed and unreflexive appraisals and processes. This article explores the governance implications of a Deliberative Mapping project that, unlike other governance principles, have emerged from an extensive process of reflection and reflexivity. In turn, the project has made significant advances in addressing the current deficit of responsibly defined criteria for shaping governance propositions. Three such propositions argue that (1) reflexive foresight of the imagined futures in which climate engineering proposals might reside is required; (2) the performance and acceptance of climate engineering proposals should be decided in terms of robustness, not optimality; and (3) climate engineering proposals should be satisfactorily opened up before they can be considered legitimate objects of governance. Taken together, these propositions offer a sociotechnical framework not simply for governing climate engineering but for governing responses to climate change at large.

## Introduction: Climate Engineering Governance

Deliberate, large-scale interventions in the Earth’s climate system have been proposed to counter anthropogenic climate change. Notwithstanding the long and checkered history of their antecedents (Fleming 2010), these climate engineering (or “geoengineering”) proposals have recently emerged as a third category of possible responses to climate change alongside reducing greenhouse gas emissions (“mitigation”) and reducing the impacts of climate change (“adaptation”). Indeed, it is the ostensible insufficiency of mitigation efforts to avoid “dangerous” climate change and of adaptive capacities to cope with a possible climate “emergency” that has fueled much of the interest in climate engineering. The term encompasses a disparate range of technology proposals that can be broadly divided into two types that seek to rectify those insufficiencies. The first type, “carbon climate engineering,” consists of proposals that seek to remove and sequester carbon dioxide from the atmosphere. The second type, “solar climate engineering,” consists of proposals that seek to reflect a fraction of sunlight away from the Earth.

Assessments of carbon climate engineering proposals have commonly found that they would be slow in their effect and expensive but pose relatively few uncertainties and risks (Vaughan and Lenton 2011). On the other hand, assessments of solar climate engineering proposals have commonly found that they would be fast in their effect and inexpensive but pose greater risks and uncertainties. However, the issues raised by climate engineering are not limited to such technical considerations but also include social and ethical concerns (Corner and Pidgeon 2010). Indeed, in its landmark report on *Geoengineering the Climate*, the UK’s Royal Society concluded that “The acceptability of climate engineering will be determined as much by social, legal and political issues as by scientific and technical factors” and that “serious and complex governance issues [would] need to be resolved if climate engineering is ever to become an acceptable method for moderating climate change” (2009, ix). The Society went on to recommend the development and implementation of frameworks for governing climate engineering research and development, and, if necessary, for eventual deployment.

Three broad approaches to governing climate engineering exist: through a multilateral organization such as the United Nations, through a consortium of states, or by lone states unilaterally (Virgoe 2009). A growing number of commentators have supported the case for governing climate engineering multilaterally through the United Nations Framework Convention on Climate Change (UNFCCC; e.g., Zürn and Schäfer 2013). This approach is advocated with particular fervor in relation to transboundary solar climate engineering proposals such as stratospheric aerosol injection (Barrett 2008). Indeed, the supposedly fast and cheap qualities of these sorts of proposals have sparked concerns over possible unilateral actions. Yet, without widespread international support, unilateral action or a simple ban consistent with the precautionary principle could be more likely to occur than multilateral decision making (Bodansky 1996). Others have dismissed fears of unilateral actions as misplaced, suggesting that international dynamics would build pressures leading to cooperation (Horton 2011). A consortium-led approach could therefore be possible and has even been advocated to avoid the lack of progress experienced in the UNFCCC negotiations (Benedick 2011). Some have suggested that regional differences in climate engineering effects would create strategic incentives to ensure that these consortia were as small as possible (Ricke, Moreno-Cruz, and Caldeira 2013).

A number of controversies relating to climate engineering experimentation, such as the UK’s Stratospheric Particle Injection for Climate Engineering (SPICE) project, the LOHAFEX (Iron Fertilization Experiment) trial (Strong et al. 2009), and the “rogue” iron fertilization of the Haida Salmon Restoration Corporation (Tollefson 2012), have recently directed governance concerns toward research as much as deployment. A number of commentators have recommended that research into climate engineering take place under the auspices of the Intergovernmental Panel on Climate Change (IPCC; Zürn and Schäfer 2013). Others, however, have observed that the “consensus science” model of the IPCC could overlook improbable but important climate engineering impacts (Victor 2008). A number of novel international institutions have also been proposed, including an International Climate Engineering Agency to coordinate and disseminate research (Zürn and Schäfer 2013) and an International Climate Engineering Research Review and Coordination Boards to coordinate climate engineering field experiments (Morrow, Kopp, and Oppenheimer 2009). Others have rejected self-regulation in favor of proactive government regulation and proposed a moratorium on large-scale climate engineering experiments but proposed experimentation thresholds below which small-scale tests might be permitted to take place (Parson and Keith 2013).

The disparate nature of the proposals encompassed by the label of “climate engineering,” however, effectively precludes the adoption of a single model for their governance. Important demarcations can be drawn between proposals with very different regulatory profiles. A distinction is often drawn between carbon and solar climate engineering proposals in that the former could largely be regulated through existing or extended legal instruments (Reynolds 2011). For example, ocean iron fertilization, a carbon climate engineering proposal, was identified as being governable through the London Convention on the Prevention of Marine Pollution by Dumping of Wastes and Other Matter (IMO 2007), a convention that has since been reratified (IMO 2013). By contrast, governance pertaining to solar climate engineering has been deemed to be largely lacking (SRMGI 2011). The jurisdictions within which climate engineering proposals may operate is another key line of distinction (Humphreys 2011). “Territorial” proposals, such as biochar and urban albedo enhancement, may be governed within the sovereign territory of states (while potentially requiring internationally collective action), whereas global “commons” proposals, such as iron fertilization and stratospheric aerosol injection, may require international agreements. There is hence the need for climate engineering governance to be sufficiently flexible to accommodate proposals under these, and other, regulatory demarcations.

Ambitions to govern climate engineering are frustrated by a much more fundamental difficulty, however: the “technology control dilemma” (Collingridge 1980). This dilemma observes that while it would be ideal to implement governance architecture in advance of significant research and development in order to guard against potentially harmful impacts, those impacts cannot be sufficiently known until that significant development has taken place. At that point, the ability to control or change a technology’s innovation trajectory becomes increasingly difficult. Flexibility is critical to overcoming the dilemma. It is with flexibility in mind that the influential, expert-defined “Oxford Principles” for climate engineering governance have been proposed (Rayner et al. 2013). This set of high-level principles argues for (1) climate engineering to be regulated as a public good, (2) public participation in decision making, (3) disclosure of research and open publication of results, (4) independent assessment of impacts, and (5) governance before deployment. Yet, some have argued that these principles, together with boundary work carried out by other academics and learned societies, have served to legitimize climate engineering research as an object of governance (Owen 2014). In doing so, they bypass “technologies of humility” that would see broad participation in their very definition (Jasanoff 2003) and implicitly reject one critical alternative pathway: that research should not be undertaken at all (see also Hulme 2014).

More recently Science and Technology Studies scholars have experimented with applying a framework for responsible innovation to climate engineering governance that would seek to manage its responsible emergence (or nonemergence) in society. Four dimensions of responsible innovation have emerged from an analysis of societal concerns raised across seventeen public engagements with emerging technologies since 2005 (Macnaghten and Chilvers 2013), including one with climate engineering (NERC 2010): anticipation, reflexivity, inclusion, and responsiveness (Owen et al. 2013). These dimensions synthesize existing calls for foresight of possible intended and unintended impacts (Ravetz 1997), institutional reflexivity (Wynne 1993), “opening up” to diversity and deliberation (Stirling 2008), and anticipatory governance (Barben et al. 2008). Indeed, this proposed framework was partly developed during efforts to instil notions of responsibility into an early climate engineering project: SPICE (Stilgoe, Owen, and Macnaghten 2013). As part of the test bed for a delivery mechanism for stratospheric aerosol injection, a technique for building responsiveness called stage gating (Cooper 1990) was used to ensure that criteria for responsible innovation were met at critical stages during research and development. As a result of the application of this technique, the test bed was initially postponed to allow for sufficient stakeholder engagement and a more thorough review of possible risks and uncertainties (Macnaghten and Owen 2011) but was later postponed indefinitely after a conflict of interest emerged concerning a patent application by one of the project investigators (Cressey 2012).

The SPICE project study in responsiveness is one of a small but growing number of studies that have sought to support the responsible innovation of climate engineering. In response to the need for more sufficient public engagement highlighted as part of the same project, other research went on to engage publics with the SPICE test bed using deliberative workshops (Pidgeon et al. 2013). Participants in that study displayed a “reluctant acceptance” of the research but stressed the need for international governance to shape future research and development. Separately, others have also engaged publics with solar climate engineering using focus groups (Macnaghten and Szerszynski 2013). Five conditions for the public acceptance of solar climate engineering emerged from these groups that demand confidence in (1) climate science as a reliable guide to policy, (2) the ability of research to anticipate side effects, (3) the ability of research to demonstrate efficacy, (4) effective governance, and (5) the capacity of democracy to accommodate solar climate engineering. The fact that framings of research and deployment were seen to be simultaneous and entangled in this study suggests that questions about governing research cannot be disaggregated from those of deployment (Owen 2014). In contrast with calls for “governance before deployment” then, it might be concluded that solar climate engineering and democracy “won’t mix” (Szerszynski et al. 2013).

Research into the responsible innovation of climate engineering has most recently sought to “open up” its appraisal in support of more reflective and reflexive governance (Stirling 2006). The “Deliberative Mapping” (DM) project has already reported on the *performance* of different climate engineering proposals in its specialist (Bellamy et al. 2013) and citizen (Bellamy, Chilvers, and Vaughan 2014) strands of engagement. This article focuses on exploring the broader and unique *governance implications* of the DM project as a whole, *analyzing* previously unexamined sociotechnical imaginaries, *synthesizing* the complete set of appraisal criteria from across both strands of engagement, *reflecting* on the performance of the process itself, and for the first time *developing* a sociotechnical framework for governing climate engineering. It begins by giving an overview of the origins and methods of the DM project, before then offering and discussing three propositions for climate engineering governance drawn from its findings. The article concludes by summarizing its contributions and posing several key recommendations for future research and policy.

## Theory and Methods: DM of Climate Engineering

Appraisals of climate engineering have begun in earnest to provide policy makers around the world with crucial decision support. A critical analysis of these appraisals (Bellamy et al. 2012), however, has revealed three significant and pervasive limitations relating to their use of framings in “closing down” policy alternatives (Stirling 2008). In turn, this risks premature entrenchment (Collingridge 1980), path dependency (David 2001), and lock-in (Arthur 1989) to particular options and future pathways (see also Cairns 2014). The first of these limitations is that climate engineering proposals have been appraised in “contextual isolation” of alternative options for tackling climate change. Two dominant problem definitions have been adopted (“insufficient mitigation” efforts and the risk of a “climate emergency”) that, taken together, marginalize legitimate mitigation and adaptation alternatives to climate engineering. The appraisals have consequently focused on assessing single climate engineering proposals or performing internal comparisons between climate engineering proposals.

The second limitation is that appraisals have largely taken the form of reductive “expert-analytic” methods such as computer modeling, cost–benefit analysis, expert review, and conventional multicriteria analysis that treat the issue as one of simple “risk” rather than one of indeterminate uncertainty, ambiguity, or ignorance (Wynne 1992; Stirling et al. 2007). While these methods have offered important insights into technical issues, they focus on narrowly framed criteria that overlook broader political, social, and ethical concerns. They have furthermore excluded the participation of wider stakeholders and publics that are normatively, substantively, and instrumentally vital for navigating the high stakes and uncertainties of post-normal science (Fiorino 1990; Funtowicz and Ravetz 1993). Of the few studies that have engaged in public participation, however, most have continued to treat climate engineering in contextual isolation; and while others have adopted broader framings, they have not treated alternative options symmetrically. Moreover, they have failed to reconcile the need for integrating mutually strengthening analytic and deliberative forms of knowledge (Stern and Fineberg 1996).

The third limitation is that appraisals of climate engineering have demonstrated little reflexivity in their acknowledgment of the framings that condition their outcomes. Ultimately, this has led to many of the appraisals prematurely “closing down” on and prescribing ostensibly preferable proposals, the foremost of which being stratospheric aerosol injection. The Royal Society’s (2009) multicriteria assessment provides a valuable illustration of this through its selection and elevation of particular criteria. Its appraisal utilized just four technical criteria: effectiveness, affordability, timeliness, and safety; of which the former two were selected to be given normative priority on the two axes of the conveying figure. Under this configuration, stratospheric aerosol injection performed the most highly, but this is the output from just one of the six possible permutations (Bellamy 2013). This need for reflexivity, joined by imperatives for diverse framings and integrated participation, compels a very particular suite of methodological responses in appraisal design (Stirling et al. 2007). One of these methods for “opening up” (Stirling 2008) is DM.

DM is an integrative analytic-deliberative multicriteria option appraisal process that engages with diverse experts and stakeholders (specialists) and lay citizens in the assessment of complex and uncertain issues (Burgess et al. 2007). It has been successfully deployed in the context of analogous issues of contested science and technology policy, including xenotransplantation (Davies et al. 2003) and radioactive waste disposal (Burgess et al. 2004). It combines the strengths of the expert-analytic Multicriteria Mapping (MCM) method (Stirling 1997) with those of the participatory-deliberative Stakeholder Decision Analysis (Burgess, Limb, and Harrison 1988), generating quantitative assessments of option performance and qualitative explorations of the reasonings underpinning such judgments. It is distinctive in that it is the only method that invites specialists and citizens to participate in the same, symmetrical appraisal process. This consistency uniquely allows for direct comparisons of convergence and divergence between different participating perspectives.

The methods used in the DM project are explained more fully in Bellamy et al. (2013) and Bellamy, Chilvers, and Vaughan (2014), but understanding the context in which the implications of the project will be discussed demands the provision of an overview here. The project took place during the summer and autumn of 2012 and comprised two parallel strands of engagement: one for specialists and one for citizens. It was framed as a study in “responding to [global] climate change” to avoid the limitations of narrower framings used in other assessments and to broaden out the context to include alternative options to climate engineering. A review of options for tackling climate change produced an extensive range of options, all of which could not be included for practical reasons. Screening the list for option diversity, seven “core” options were selected to be appraised by all participants in the study, and seven “discretionary” options were made available for appraisal by participants at their discretion. Participants were also free to appraise their own “additional” options. Table 1 shows definitions for the core, discretionary, and commonly appraised additional climate engineering proposals included in the project (see Bellamy et al. [2013] and Bellamy, Chilvers, and Vaughan [2014] for definitions of the full list of options included in the project).

**Table 1.** The Definitions of “Core” Climate Engineering Options (C1–3) Appraised by All Participants, “Discretionary” Climate Engineering Options (D1–3) Appraised by Some Participants at Their Discretion, and a Commonly Appraised “Additional” Climate Engineering Option (A1) Appraised by Participants at Their Initiative.

Proposal	Definition
C1 Biochar	Focusing research and development into the production of biochar and its application to soils.
C2 Air capture and storage	Focusing research and development into the use of technology for capturing CO_2_ from the ambient air.
C3 Stratospheric aerosol injection	Focusing research and development into the injection of reflective sulfate particles into the stratosphere.
D1 Iron fertilization	Focusing research and development into the application of iron to the ocean to stimulate algal growth.
D2 Cloud albedo enhancement	Focusing research and development into the use of technology to enhance cloud reflectivity.
D3 Space reflectors	Focusing research and development into the use of reflective mirrors in Earth orbit.
A1 Afforestation	Increasing the proportion of the Earth’s land surface covered by forests.

The participants themselves were selected from wider groups of candidates to represent a diversity of perspectives. For the specialist strand, twelve participants with seniority and an appreciation of the international context of climate change were recruited from across four sectors: academia, civil society, industry, and government. Disciplinary perspectives on climate engineering proposals, mitigation options and adaptation from across the natural and social sciences were represented, as were opposing attitudes to conducting climate engineering research. For the citizen strand, thirteen participants were recruited to be sociodemographically representative of the area in which the engagement would take place: Norfolk (UK). Different perspectives on priorities for social concern were represented, as were those on the causes of and solutions to global environmental issues.

Both the specialist and citizen strands followed the same, four-step multicriteria option appraisal process in which participants (1) selected and defined options to appraise, (2) developed a set of criteria with which to appraise those options, (3) scored the relative performance of the options against those criteria, and (4) gave weightings to the criteria to indicate their relative importance. For the specialist strand, this process was conducted in two one- to three-hour face-to-face MCM interviews for each participant. For the citizen strand, it was conducted through two full-day reconvened citizens’ panel workshops stratified by gender. The development of criteria was thus an individual undertaking for the specialists, whereas for the citizens it was one of group-based negotiated amalgamation. The two strands came together in a “joint citizen–specialist workshop” halfway through the process, where citizens were encouraged to challenge expert views in an inversion of usually accepted power relations.

## Results and Discussion: Propositions for Governing Climate Engineering

The resultant performances of different options for tackling climate change arising from the DM appraisals are described more fully in Bellamy et al. (2013) and Bellamy, Chilvers, and Vaughan (2014), but it is in this article that their implications for governing climate engineering are synthesized and discussed. To this end, it will be necessary to briefly elucidate the project’s main findings. In “opening up” climate engineering for the first time, the DM project generated radically different results to those of previous assessments. These included (1) a significant expansion of criteria “depth”: the diversity of alternative criteria that pertain to an ostensibly discrete criterion, such as “efficacy”; (2) a significant expansion of criteria “range”: the diversity of alternative criteria at large, such as the inclusion of more “social” criteria as well as “technical” criteria, giving rise to a very different view of option performance where climate engineering proposals were outperformed by mitigation alternatives; and (3) validation in resituating climate engineering proposals within the broader context of alternative options for tackling climate change and of opening up their appraisal more broadly.

These findings pose correspondingly unique implications for climate engineering governance, that unlike other proposed governance recommendations have emerged from an extensive process of reflection and reflexivity (Stirling 2006). In turn, the DM project has made significant advances in addressing the current deficit of responsibly defined criteria for shaping governance propositions (Owen 2014). This section outlines and discusses three such propositions that, taken together, offer a framework not simply for governing climate engineering but for governing responses to climate change at large. Given the focus of this article, however, the propositions will be discussed here with particular attention to climate engineering. It is not a “technoscientific” framework in that it does not encompass only the visionary thoughts of (natural or social) scientists (Marcus 1995) but “sociotechnical” through its recognition of “how, through the imaginative work of varied social actors, science and technology become enmeshed in performing and producing diverse visions of the collective good” (Jasanoff 2015, 15). Crucially, unlike other principles for governing climate engineering, the propositions do not preclude the possibility for research not being undertaken at all.

### Imaginaries, Foresight, and Reflexivity

“Collectively imagined forms of social life and social order reflected in the design and fulfilment of scientific and/or technological projects” (Jasanoff and Kim 2009, 120) constitute important influences on the relationship of prospective sciences and technologies to political power. Such “sociotechnical imaginaries” of the futures within which climate engineering proposals might reside were elaborated through the appraisal criteria developed in the DM project. As the driving forces behind the envisaged purposes of climate engineering, criteria pertaining to the appraisal of option efficacy demand special attention here. In expanding the depth of efficacy criteria beyond those used in previous assessments, five distinct sociotechnical imaginaries for climate engineering emerge from thirteen discrete efficacy criteria developed in the project (see Table 2). These imaginaries are in evidence at different instants of coproduction, undergoing emergence, contestation, or stabilization (Jasanoff 2004).

**Table 2.** Sociotechnical Imaginaries for Options for Responding to Climate Change.

Imaginary	Description^a^
Supplement mitigation	Options that reduce or stabilize greenhouse gas emissions or atmospheric concentrations of carbon dioxide will strengthen conventional mitigation efforts and help avoid “dangerous” climate change (CO_2_ concentration stabilization, emissions reduction, and greenhouse gas reduction)
Supplement adaptation	Options that weaken the impacts of climate change will strengthen conventional adaptation efforts and help protect against those changes that cannot be mitigated against (climate change impacts reduction)
Climate emergency	Options with a fast climatic response time will substitute for insufficient conventional mitigation and adaptation efforts in the face of a sudden climate change “emergency” (climatic response time)
Global thermostat	Options that impact on or reduce global temperature or seek to maintain a constant global temperature will regulate the climate (global warming reduction, global temperature maintenance, and impact on global warming)
Sustainable reliability	Options with little uncertainty and a sustainable duration of effect will protect the climate in the long term (duration of effect and efficacy uncertainty)

^a^Efficacy criteria pertaining to each corresponding imaginary are given within parentheses. Three criteria are not shown. Appraising the “efficacy and completeness” of an option refers to an option’s capacity to respond to the suite of problems posed by climate change, and therefore subsumes each imaginary. Appraising the “scale of effectiveness” and “efficacy of intended impacts” refers to an option’s performance on its own imagined terms.

One of these imaginaries, of a future in which climate engineering proposals might supplement mitigation efforts, maps directly upon a dominant problem definition identified as having framed other climate engineering appraisals: the “insufficient mitigation” frame (Bellamy et al. 2012). This imaginary is not greatly contested among scientists or stakeholders alike, who increasingly recognize that “dangerous” climate change beyond 2°C may not be avoided without the use of (carbon) climate engineering (McLaren 2012). Indeed, the use of at least two carbon climate engineering proposals (large-scale afforestation and bioenergy with carbon sequestration) is already assumed in the representative concentration pathway scenarios used for the IPCC’s Fifth Assessment Report (see van Vuuren et al. 2011). A second imaginary, in which climate engineering proposals might supplement adaptation, is similarly uncontested. It extends the mitigation imaginary in anticipating a future where none of the three main categories of climate change response will be sufficient in isolation. Indeed, some have called for a combined mitigation, adaptation, and geoengineering approach to climate policy (IMechE 2009).

A third imaginary, in which mitigation and adaptation could be wholly ineffectual, details a climate change “emergency,” which may only be overcome with (solar) climate engineering. This maps upon another dominant problem definition identified as having framed other climate engineering appraisals (Bellamy et al. 2012). What is curious here is that both of these dominant definitions arose independently of each strand in the DM project and without having been introduced by the research team, suggesting a saliency of these imagined futures around which climate engineering proposals are being built. Unlike the mitigation imaginary, however, that of the climate emergency is highly contested outside of its small community of proponents. The notion that solar climate engineering proposals could be deployed preemptively or in response to an emergency rests upon two critical assumptions. First, preemptive deployment would require reliable “early warning,” for which the possibility is limited due to noise (e.g., Ditlevsen and Johnsen 2010; cf. Lenton 2011). Second, to paraphrase Mike Hulme, that “emergencies are declared, not discovered” (Hulme 2014, 134) means acceptance for responsive deployment can never be “objective” and is thus unlikely to be shared.

A fourth imaginary evokes a related but more overtly hubristic future where the development of a global thermostat is sought to regulate the climate. This highly contested imaginary is one that is prevalent in debates around climate engineering and in particular around solar climate engineering and stratospheric aerosol injection. Hulme (2014, 54) argues that “the idea that global temperature is a suitable object of governance and one through which the well-being of humanity can be secured is a delusion.” He argues that such an endeavor conflates regional and local peoples and climates and would be both ungovernable and unreliable. Indeed, he describes stratospheric aerosol injection as “a flawed idea … that seeks an illusory solution to the wrong problem” (Hulme 2014, 130). But sustainable reliability is a fifth imaginary in which options for tackling climate change will be controllable, long-term undertakings: a vision that is very much at odds with critics of the global thermostat.

These five imaginaries begin to reveal the imagined futures around which the purposes of climate engineering are being constructed. Just as Selin (2007) has argued in relation to expectations for nanotechnologies, “there is little innocence in such imaginings.” The collective visions compete in a “battle for power over the future” (Selin 2007, 215), forcefully influencing social responses to innovation (Jasanoff and Kim 2013). The imagined futures seen here and those yet to emerge cannot be *predicted*, however, but they can be *anticipated* in more inclusive, democratic ways (Guston 2013). As well as through the DM approach taken here, policy makers and other actors can support such anticipatory governance through deliberative methods, scenario workshops, or vision assessments (see Barben et al. 2008). In doing so, participants should engage not only in self-referential critique but in wider institutional reflexivity where the assumptions and commitments that shape governance itself are scrutinized (Wynne 1993). In other words, policy makers and other social actors must gain reflexive foresight of the sociotechnical futures in which climate engineering could reside.

### Performance Optimality and Robustness

“Optimal” solutions, by definition, should be the best courses of action available to decision makers. Yet, in reality, uncertain or ambiguous outcomes often render these pathways undesirable. Indeed, we have already seen how narrow and unreflexive framings in climate engineering assessment give rise to ostensibly “optimal” solutions to climate change (Bellamy et al. 2012). When those framings are uncovered, however, it becomes clear that optimal solutions are only optimal under very specific conditions. An answer to this optimality paradox has its origins in the field of operational research: robustness (Rosenhead, Elton, and Gupta 1972). Rather than seeking optimal solutions, it is argued, decision makers should look for robust ones. The idea of “robustness” began as “a measure of the flexibility which an initial decision of a plan maintains for achieving near-optimal states in conditions of uncertainty” (Rosenhead, Elton, and Gupta 1972, 413). The concept of robustness has since been developed for identifying options under deep uncertainty that perform “relatively well, compared to alternatives, across a wide range of plausible futures” (Lempert et al. 2006, 514) and has been encouraged in climate change adaptation policy (Dessai et al. 2011). It more broadly highlights the need for developing “socially robust knowledge” that is tested for validity “outside the laboratory,” engages with extended expertise, and is continually tested, expanded, and modified (Nowotny, Scott, and Gibbons 2001).

This article further develops the concept of robustness to mean *the identification of sociotechnical pathways that perform relatively well, compared to alternatives, under a diversity of reflexive framings*. Simply put, the more diverse the framings that bear on an appraisal, the more robust any conclusions that are drawn from it will be. In turn, options that perform well under these framings can be considered more robust, and so too can decisions on how to govern them. In the DM project, diverse academic, civil society, industry, government, and public perspectives independently developed eighty discrete criteria with which to appraise climate engineering proposals and other options for tackling climate change. These criteria comprise thirty-nine criteria subgroups and nine criteria groups (see Table 3). These groups span technical issues of efficacy, environment, feasibility, economics and safety, and social issues of politics, society, ethics, and co-benefits. For an option to be considered robust, it should thus perform not only relatively well against one discrete criterion but also against other discrete criteria within a criteria subgroup and against discrete criteria within criteria subgroups of the other criteria groups. Moreover, this performance should be relatively good not only against one discrete perspective but also against other perspectives of the same and different classifications.

**Table 3.** Criteria for Appraising Options for Tackling Climate Change.

Criteria Group	Criteria Subgroups	Discrete Criteria
Efficacy	Climate change impacts reduction,^a^ climatic response time,^a^ duration of effect, efficacy of intended effects, global temperature reduction,^a^ and greenhouse gas reduction^a^	Climate change impacts reduction,^a^ climatic response time,^a^ CO_2_ concentration stabilization,^a^ duration of effect, efficacy and completeness,^a^ efficacy of intended impacts, efficacy uncertainty,^a^ emissions reduction,^a^ global temperature maintenance, global warming reduction, greenhouse gas reduction, impact on global warming,^a^ and scale of effectiveness
Environment	Carbon footprint, environmental impacts,^a^ environmental side effects,^a^ impact reversibility, and transboundary impacts^a^	Carbon footprint, environmental impacts, environmental risk, foreseeable environmental impacts, impact reversibility, risk of adverse effects, environmental safety,^a^ transboundary effects,^a^ unforeseen impacts, unintended consequences, unintended environmental impacts,^a^ unintended environmental risks, and unintended or unanticipated risks
Feasibility	Development time, resource availability, state of knowledge, and technical feasibility^a^	Demonstration, ease of operation, ensemble uncertainty, feasibility, lead time, practicality, scalability, technical feasibility,^a^ technical know-how, and workability
Economic	Commercial viability, cost,^a^ cost–benefit ratio, cost-effectiveness, economic sustainability, investment return, and public investment	Affordability, cost–benefit ratio, cost,^a^ cost-effectiveness, economic cost,^a^ economic efficiency, economic feasibility, investment return, public investment, set-up cost, subsidization, and sustainability
Political	(Inter)governmental cooperation, governance,^b^ political acceptability,^a^ and political viability	Democratic compatibility^a^; (inter)governmental cooperation; governance^b^; legislation; political acceptability^a^; political feasibility; political, social, and legal feasibility; political and technical feasibility; and political will
Safety	Impacts on humans^b^ and side effects on humans	Dangerousness, human impacts,^b^ and side effects on humans
Social	Cultural acceptability, social acceptability,^a^ and socioeconomic impacts	Cultural acceptability, impact on lifestyles, public acceptability,^a^ public convincement, public perception, social acceptability, social support, and socioeconomic impacts^a^
Ethical	Availability, distributive justice,^a^ ethical questions, intergenerational equity,^b^ misuse, morality, and ownership and control^a^	Availability, centralized or distributed control,^a^ ethical questions raised, fairness in practice, intergenerational equity,^b^ monitoring; moral obligation, moral pursuit, openness to abuse, social impact progressivity,^a^ and social inequality reduction
Co-benefits	Co-benefits^a^	Co-benefits^a^

*Note:* All listed are criteria except where ^a^ indicates that the corresponding criterion was also used as a principle to rule options out, and ^b^ indicates a principle.

Unlike other methods of participatory appraisal that seek to build a consensus between different perspectives on an issue, DM is designed to map the divergence of those perspectives. It is especially remarkable then that the findings of the project revealed a high degree of consistency between diverse perspectives on climate engineering proposals and other options for tackling climate change (see Bellamy et al. [2013] and Bellamy, Chilvers, and Vaughan [2014] for detailed analyses of option performance). With the exception of the participating industry specialists, climate engineering proposals were consistently outperformed by mitigation option alternatives. Three tiers of performance emerged (see Box 1), with voluntary low carbon living, offshore wind energy, and afforestation constituting the “highest” performing options. In contrast, climate engineering proposals constituted three of the four “lowest” performing options: stratospheric aerosol injection, iron fertilization, and space reflectors. The expected likelihood of the fourth lowest option, business as usual, being deliberately, tacitly, or inadvertently pursued, compels adaptation as a priority response to its myriad harmful impacts documented across the criteria groups. The eight remaining “middle” options (a new carbon market mechanism, biochar, air capture and storage, nuclear fusion energy, nuclear fission energy, coal energy with carbon capture and storage, a carbon tax, and cloud albedo enhancement) displayed a more ambiguous performance.

Box 1. **Three Tiers of Performance among Options for Tackling Climate Change.^a^**

The performance of three “highest” ranking options can be considered more robust. *Voluntary low carbon living* ranked highest in both citizens' panels and for the specialists as a whole, and for the civil society and government sectors. However, one industry specialist ruled this option out on feasibility grounds. *Offshore wind energy* ranked second highest in both panels of citizens and for the specialists as a whole, and it ranked highest for the academic sector. *Afforestation* (DC) ranked third highest in both panels of citizens. The option was also advanced as an additional option by an academic specialist, where the option too scored highly.

The performance of eight “middle” ranking options can be considered more ambiguous. A *new market mechanism* ranked moderately with the male citizens' panel and poorly with the female panel, but third highest for the specialists as a whole, and moderately across the sectors. However, one civil society specialist ruled this option out on efficacy, environment, social, and ethical grounds. *Biochar* ranked third highest for both panels of citizens, but moderately overall and for the specialists as a whole, and across the sectors. *Air capture and storage* ranked poorly with the male citizens' panel and moderately with the female panel, and moderately for the specialists overall, and it ranked highest for the industry sector. *Nuclear fusion energy* (DC, DS3) ranked highly for both citizens' panels and moderately for the specialists, toward the lower end of the “middle” ranking discretionary options. *Nuclear fission energy* (DS3) ranked moderately for the specialists. *Coal energy with carbon capture and storage* (DS3) ranked moderately for the specialists, toward the higher end of the “middle” ranking discretionary options. A *carbon tax* (DS4) ranked moderately for the specialists, toward the higher end of the “middle” ranking discretionary options. *Cloud albedo enhancement* (DS4) ranked moderately for the specialists.

The performance of the remaining four “lowest” ranking options cannot be considered robust. *Stratospheric aerosol injection* ranked third lowest in both panels of citizens and second worst for the specialists as a whole, and for the specialist sectors, except where it ranked worst for the industry sector. Two academic specialists, one civil society specialist and one government specialist ruled this option out on efficacy, environment, political, social, ethical, and co-benefits grounds. *Business as usual* ranked lowest in both panels of citizens and for the specialists as a whole, and for the specialist sectors, except where it ranked second lowest for the industry sector. One academic specialist, two civil society specialists, and two government specialists ruled this option out on efficacy, environment, social, and ethical grounds. *Iron fertilization* (DS5) ranked lowest of the discretionary options for specialists. One civil society specialist ruled this option out on efficacy, environment, social, and ethical grounds. *Space reflectors* (DS3) ranked second lowest of the discretionary options for specialists. One academic specialist ruled this option out on economic grounds.

^a^Options that were not appraised by all participants in the DM project are followed by caveats in parentheses: (DC) indicates a discretionary option appraised by all citizens and (AC) indicates an additional option appraised by all citizens and (DS#) indicates a discretionary option appraised by a specified number of specialists. Additional options that were appraised by sole participants are not included here.

The highest performing options can hence be considered more robust, having performed relatively well against the diversity of criteria groups and perspectives. Adaptation, too, can be considered more robust, through its broad capacity for responding to the impacts posed by business as usual across the criteria ensemble. Of course, these options are by no means optimal. For example, while voluntary low carbon living performed relatively highly against most criteria groups, against efficacy and co-benefits criteria, it performed relatively poorly. Thus, it is clear that no single option for responding to climate change is a panacea, reaffirming assertions of there being no elegant “silver bullet” (Prins and Rayner 2007), still less from climate engineering (Shepherd 2012). Yet, it is nevertheless clear that some options are most robust than others, which in turn poses implications for resource allocation. This is not to suggest that research into less robust climate engineering proposals should be abandoned or that any specific proposals can be yet ruled out (although there were perspectives in the DM project that did rule out certain options on principle, most often stratospheric aerosol injection and business as usual). Instead, resources should be allocated proportionally according to different options’ robustness. Mitigation options and adaptation are therefore the relative priority, which would concurrently go some way toward alleviating concerns over a “moral hazard” whereby those efforts might otherwise be diminished (Royal Society 2009).

### Responsibility, Legitimacy, and Flexibility

By resituating climate engineering proposals in the broader context of alternative options for tackling climate change, by broadening out inputs from diverse perspectives and appraisal criteria, and by reflexively opening up uncertainties, ambiguities, and ignorance, the DM project has acted to guard against premature entrenchment, path dependency, and lock-in. Yet, making decisions based on the findings of any research inevitably requires some degree of closure, precluding opportunities for the elicitation of further alternatives, perspectives, and forms of incertitude. Two key questions thus arise for how to go about governing climate engineering. The first is temporal: when should such closure take place? In other words, can climate engineering proposals ever be considered legitimate objects for governance (Owen 2014)? The answer to this question lies with how satisfactorily governance propositions can be responsibly (anticipatorily, inclusively, reflexively, and responsively) defined.

In supporting dimensions of responsible innovation, the DM project can offer some valuable insights for the question of object legitimacy. It shows that particular climate engineering proposals are indeed legitimate objects for governance. These are proposals that have demonstrated relatively higher levels of robustness and would impose their effects on relatively fewer people, relative to the scales at which they could be used (see Bellamy et al. [2013] and Bellamy, Chilvers, and Vaughan [2014] for detailed analyses of proposals’ impacts). Afforestation, biochar, and air capture and storage, for example, each threaten to impose some risks, including loss of species biodiversity, changes to soil quality, and instability of geological storage reservoirs, respectively. However, as land-based proposals such risks are localized and not intrinsically transboundary in nature. This leads to the second question, which follows from the first and is procedural: if seen to be satisfactorily legitimate objects for governance, *how* can climate engineering proposals be governed? For these above proposals, self-regulatory or “soft” regulatory measures may be most appropriate in early stages of research. These might include responsibly defined codes of practice drawn up from processes such as DM. From this project, we have already seen codes for foresight and reflexivity, and robustness not optimality.

There are, however, climate engineering proposals that have demonstrated relatively low levels of robustness and would impose their effects on many people relative to the scales at which they could be used. Stratospheric aerosol injection, iron fertilization, cloud albedo enhancement, and space reflectors, for example, threaten to impose a sudden cessation “termination problem,” monocultures and dead zones, interference with regional weather patterns, and disruptions to global circulation, respectively. As atmospheric-, oceanic-, or space-based proposals such risks are inherently international and transboundary or even global in nature. The DM project finds the legitimacy of these proposals as objects of governance, in agreement with Owen (2014), to be unresolved questions. While the DM project has undoubtedly introduced governance propositions that are more responsibly defined than those of other expert-defined recommendations, it is limited by its modest scale, involving twelve international specialists and thirteen UK citizens. For the above proposals, a more internationally diverse engagement is imperative for any governance propositions to be considered as even remotely legitimate. In short, more “opening up” is required.

Nevertheless, field experiments with risky climate engineering proposals have already taken place (e.g., Strong et al. 2009; Cressey 2012; Thiele et al. 2012) or are planned for the future (e.g., Latham et al. 2012; Dykema et al. 2014; Keith, Duren, and MacMartin 2014). Even efforts to reign in such experiments with regulatory regimes have been met with limited success. Experiments in iron fertilization, for example, have repeatedly, but ambiguously, violated soft international treaties (Tollefson 2012). It may thus become necessary to strive for effectively governing these proposals even in the absence of responsibly defined governance propositions. A common way of regulating novel technologies is by setting thresholds to restrict experimental scales to levels that can be considered “safe.” Indeed, Parson and Keith (2013) proposed a threshold for stratospheric perturbation experiments of ΔRF < ∼10^−6^ W m^−2^, below which research could proceed. Yet, appeals to safety are to miss the point that it is broader political, social, and ethical concerns that make these proposals so contentious. As Lee and Petts (2013) argue, for unresolved “questions regarding the appropriateness of safety thresholds, it may be more appropriate to act in a precautionary manner. This will certainly be so where a credible threat has been identified, even if its scope and impact is scientifically uncertain” (Lee and Petts 2013, 155). It might be then that “hard” regulatory measures may be best placed for governing such proposals in the early stages of research. These might include command-and-control measures such as moratoria.

Yet, any regimes that are adopted for governing climate engineering proposals must remain flexible and adaptive. As more opening up occurs, governance propositions will become more responsibly defined and socially legitimate, and as new scientific information becomes available, technical risks may become better characterized. Regulatory regimes should therefore be reviewed in tandem with developments in the research and innovation process (Frater et al. 2006). There are limits, however, to the adaptability of innovation governance (Lee and Petts 2013), that pose particular challenges for climate engineering. First is the limitation of scale. For those proposals with truly international or global governance implications, it may prove difficult to reach agreement. Second is the limitation of speed. The ostensible risk of a climate emergency gives new meaning to the “tyranny of urgency” that threatens innovation going “out of control” (Lee and Petts 2013, 159), providing renewed pressure for moratoria, or at the very least mechanisms for slowing the pace of innovation, such as stage gating (Cooper 1990). The third relates to public participation. Those proposals with international or global governance implications make the possibility for meaningful engagement, and by extension legitimate governance, vanishingly improbable.

## Conclusions

The DM project has begun to address the current deficit of responsibly defined criteria for shaping governance propositions for climate engineering. Unlike other governance principles, they have emerged from an extensive process of reflection and reflexivity (Stirling 2006), engaging with diverse experts, stakeholders, and citizens, and ultimately opening up alternative pathways for responding to climate change. The propositions that arise from this process are thus central not only to the good governance of climate engineering proposals but to options for responding to climate change at large. They argue that (1) reflexive foresight of the imagined futures in which climate engineering proposals (and other options for tackling climate change) might reside is required; (2) the performance and acceptance of climate engineering proposals (and other options for tackling climate change) should be decided in terms of robustness, not optimality; and (3) climate engineering proposals (and other options for tackling climate change) should be satisfactorily opened up before they can be considered legitimate objects of governance.

The resultant sociotechnical framework for governing options for tackling climate change supports wider ambitions for the responsible innovation of emerging sciences and technologies (Owen et al. 2013). Its constituent propositions each call for the anticipation of possible impacts, deep reflexivity, inclusive deliberation, and responsiveness to changing states of knowledge and societal values. The framework is thus equally central to the good governance of any emerging science or technology. Gaining reflexive foresight of the imagined futures in which technoscientific projects may reside will engender more democratic responses to governing their innovation. Judging the performance of alternative options for responding to a problem in terms of robustness rather than optimality will allow us to base decisions of acceptance or rejection on more thorough and complete information. Ensuring the satisfactory opening up of technoscientific projects will help us to responsibly define criteria for their governance, and whether they are, in point of fact, legitimate objects of governance in the first instance. “Opening up” is a foundational approach to addressing the challenges of twenty-first century innovation, which will track the evolution of this framework as it flexes and adapts to changing states of knowledge.
